# Successful laparoscopic trans-peritoneal repair of an incisional inguinal hernia, resulting from deep lymph node dissection for melanoma: A case report

**DOI:** 10.1016/j.ijscr.2020.01.019

**Published:** 2020-01-23

**Authors:** M. Clementi, M. Di Furia, F. Sista, A.R. Mackay, S. Guadagni

**Affiliations:** Department of Applied Clinical Sciences and Biotechnology, University of L’Aquila, 67100 L’Aquila, Italy

**Keywords:** Deep groin lymph-node dissection, Melanoma, Incisional inguinal/groin hernia, Laparoscopic trans-peritoneal repair

## Abstract

**Introduction:**

Deep pelvic lymph node dissection for cancer may result in incisional inguinal hernias. We present a case report of successful laparoscopic trans-peritoneal repair of a large ventral inguinal hernia that developed following ileo-inguinal lymph node dissection (CLND) for melanoma.

**Case presentation:**

A successful 3 port laparoscopic trans-peritoneal procedure was performed on a 56-year-old female for the repair of a left inguinal hernia, developed 13 months following CLND for melanoma. The large oval 18 × 14 cm inguinal defect, with superior margins bordering the conjoint tendon and inferior margins bordering the ileo-psoas muscle, femoral vessels and nerve, was not closed in order to avoid excessive tension and was repaired by fixing a 25 × 20 cm intra-peritoneal mesh to abdominal borders at superior and lateral margins with permanent fasteners and at the inferior margin by a cyanoacrylate-glued overlap to protect femoral vessels and nerves from damage. No hernia recurrence was observed 8 months following this procedure.

**Discussion:**

Incisional inguinal hernias, following CLND, are rare but present a challenge to surgeons due to the difficulty in identifying both anatomical plains and safe sites for stable repair.

**Conclusions:**

We report a laparoscopic trans-peritoneal approach for the safe, reproducible and efficacious repair of incisional inguinal hernias that result from CLND. In our opinion prevention of hernia recurrence can be achieved by a intraperitoneal large mesh fixed at superior and lateral margin borders with permanent fasteners and using cyanoacrylate glue to overlap inferior margin borders in order to prevent vessels and/or nerve injury.

## Highlights

• Groin incisional hernia may result like late complication of deep pelvic dissection.
• When this type of inguinal ventral hernia develops, the surgeon is facing some problems different to the common ventral hernia repair.
• Most of these difficulties may be overcome using laparoscopic approach.
• We present a case of successful laparoscopic repair of a giant ventral hernia developed like late complication of deep pelvic dissection for melanoma.
• To our knowledge, no other laparoscopic repair of this type of ventral hernia has been previously reported.

## 1 Introduction

For many years, completion ilio-inguinal lymph node dissection (CLND) has been the cornerstone for managing sentinel lymph node biopsy (SLNB)-positive stage 3 melanomas and therapeutic LN-dissection (TLND) recommended for patients with suspected lymph node involvement.

Ilio-inguinal lymph node dissection associates with a high rate of early postoperative complications, with 20–77% of patients developing skin necrosis, wound dehiscence, seroma and lymphocele [[1], [2], [3]]. Furthermore, the section of anterolateral abdominal wall muscles along the iliac crest and inguinal ligament lateral to the femoral artery, required for extraperitoneal pelvic lymph node dissection, may also result in insertional ventral inguinal hernia, as a late development.

Traditional repair of incisional inguinal hernias with mesh, is challenging due to difficulty in identifying appropriate safe anatomical regions for strong and stable mesh anchoring, with few reports concerning this complication and no standardized procedures for repair.

To this end, we present a case report of successful laparoscopic repair of a large ventral inguinal hernia in a 56-year-old female who developed an incisional inguinal hernia, as a late complication of ileo-inguinal CLND for melanoma. To our knowledge, this represents the first report of successful repair of this type of hernia. We discuss the technical difficulties encountered during this procedure and the potential advantages of this approach, in this report, in line with SCARE criteria [4].

## 2 Case presentation

**Fig. 1:**
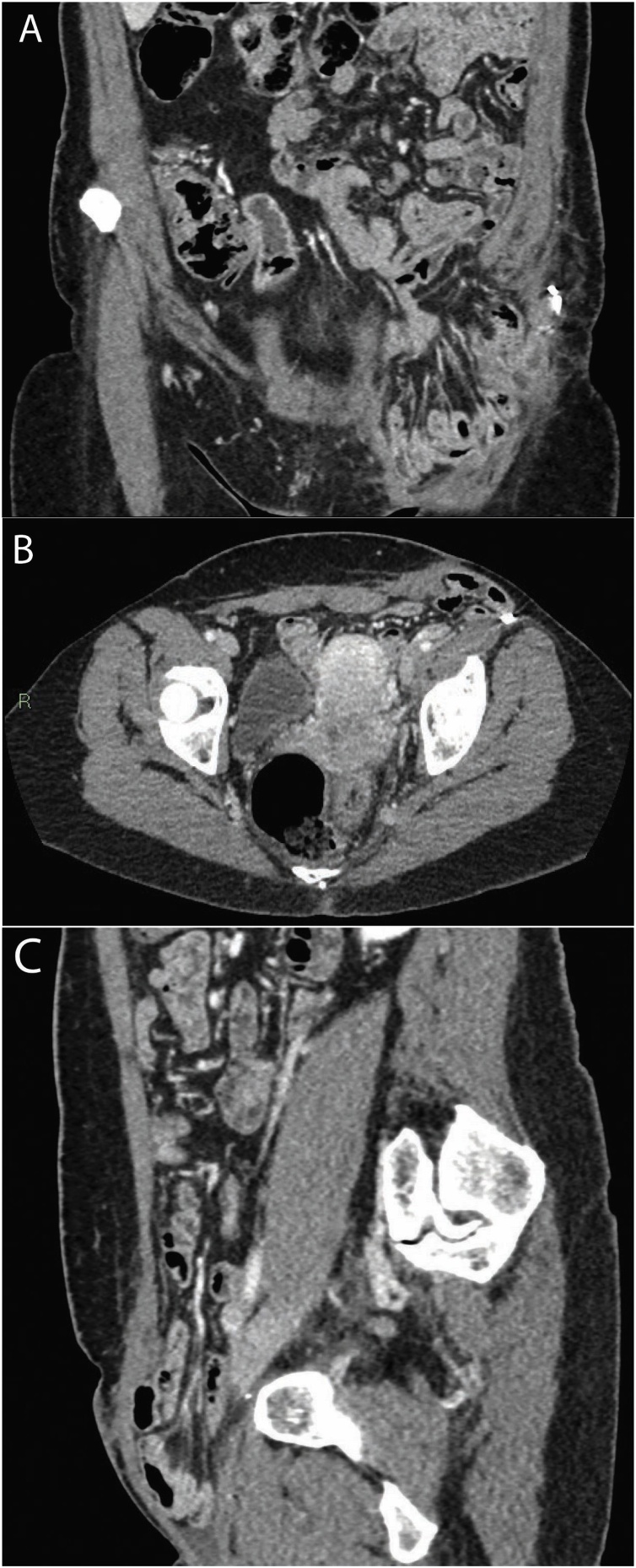
CT scan demonstrating a large incisional inguinal hernia, with clustered small bowel loops descending into the groin area. Coronal (A), transversal (B) and sagittal (C) sections.

The 56-year-old female patient underwent left side ilio-inguinal CLND, following SLNB confirmation of lymph node metastasis from a thick homolateral-thigh malignant melanoma. The ilio-inguinal CLND procedure, performed in another hospital, involved inguinal ligament section for complete deep iliac space lymph-node dissection. The patient was subsequently referred to our Surgical Unit with post-operative persistent seroma, which was treated by percutaneous drainage for 3 weeks to reduce daily serous lymphatic fluid output to <80 ml and then by 4 ultrasonic guided aspirations, performed in the following 3 weeks. The patient also developed lymphoedema of the leg, which was treated by physiotherapy and compression garments. Five months following CLND, the patient developed a painful inguinal swelling, which was diagnosed by CT scan as a large incisional inguinal hernia (Fig. 1). A laparoscopic trans-peritoneal hernia repair procedure was programmed and the patient, informed of the risks and advantages, provided full written consent.

### 2.1 The surgical procedure

**Fig. 2:**
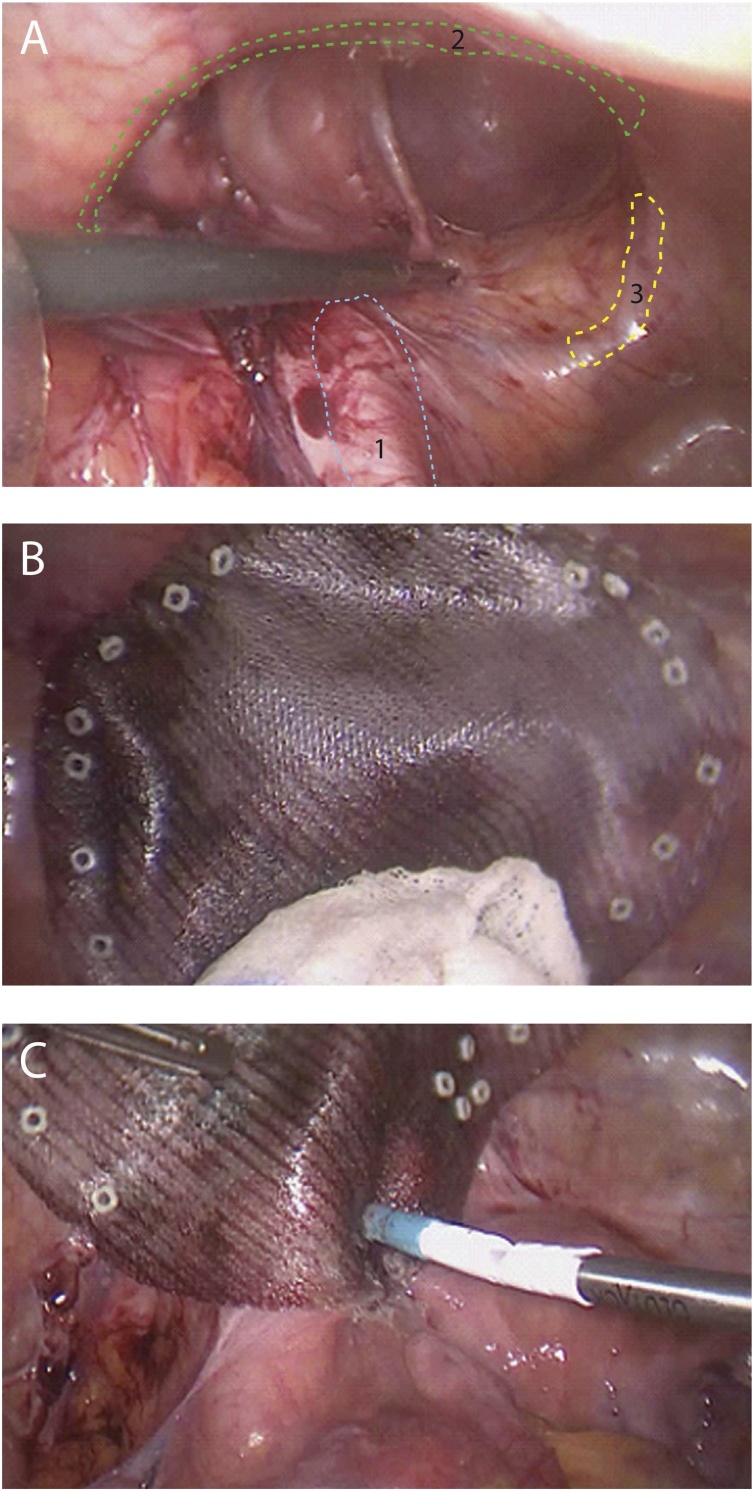
A. Appearance of the parietal defect after adhesiolysis: 1 – iliac vessels, 2 – residual conjoint tendon, 3 – pubic tubercle. B. Superior and lateral mesh fixation with permanent fasteners. C. Inferior mesh fixation with cyanoacrylate glue.

The patient, who was melanoma disease-free at 1-year follow-up, was subjected to ventral hernia repair, 13 months following CLND. The surgeon, with adequate experience in laparoscopic repair of ventral and inguinal lesions [5], performed a 3 port laparoscopic procedure with the patient in Trendelenburg’s position. Briefly, a 10–12 mm camera port (also utilized for prosthetic mesh insertion) was positioned in the midline 2 cm above the umbilicus and two additional 5 mm ports inserted in the left flank and right iliac fossa. Adhesiolysis of small bowel loops and the hernial sac was very carefully performed by blunt dissection to avoid enterotomies. A large oval 18 × 14 cm defect was identified, the superior margins of which bordered the conjoint tendon, inserted laterally to the anterior superior iliac spine and medially to the tuberculum pubis, and inferior margins of which bordered the ileo-psoas muscle, femoral vessels and nerve (Fig. 2a). The hernial sac was not removed due to its tight adhesion to a cutaneous scar and was not closed (sutured) in order to avoid excessive tension at its margins. The 25 × 20 cm mesh (Ventralight™ ST Mesh, Bard) used for repair, guaranteed a minimum 3 cm overlap of lesion margins, was fixed to the abdominal wall at the superior and lateral margins with permanent fasteners (CapSure™ Permanent Fixation System, BARD) (Fig. 2b) and glued at the inferior lesion margin with cyanoacrylate glue (Glutack® 50, Glubran 2, GEM) in order to create a 5 cm overlap of femoral vessels and psoas muscle to prevent femoral vessel and/or nerve injury (Fig. 2c), and surgery was completed within 65 min. The post-operative course was uneventful, the patient discharged on day 3. At 8 months the patient did not exhibit any clinical or radiological signs of hernia recurrence and at 2 years follow-up remains melanoma-free.

## 3 Discussion

Regional inguinal lymph node metastases are not infrequent in stage 3 melanoma patients with primary tumours in the lower extremities and/or trunk. Although approaches for regional nodal basin management continues to evolve in these patients [6], completion ilio-inguinal lymph node dissection remains a widely recognised strategy for clinically and SLNB confirmed metastases. Inguinal dissection can be superficial to remove all femoral and inguinal lymph nodes (Inguinal dissection) or extended to lymph nodes within the deep pelvic iliac space, including iliac lymph nodes up to iliac artery bifurcation and obturator lymph nodes, with extent of dissection determined by SLNB, clinical evaluation and/or pelvic imaging [7].

Excluding laparoscopic or robotic ileo-inguinal LND [8,9], different technical approaches have been reported for traditional CLND to reduce morbidity rates, including: skin incision type; a thick skin flap; saphenous vein preservation; sartorius muscle repositioning over femoral vessels; inguinal ligament section and skin trimming during closure [[10], [11], [12]]. Once decided, traditional surgical approaches for ilioinguinal dissection involve sectioning the inguinal ligament and anterolateral abdominal wall muscles, lateral to the femoral artery. This greatly improves exposure and subsequent lymph node dissection, permitting the en-bloc dissection of all superficial and deep lymphatic tissues. The main potential disadvantage of this approach is long-term abdominal wall weakness, leading to abdominal incisional hernia. However, to date few reports have dealt with the risk of developing inguinal ventral hernias as a result from these procedures and subsequent complications in terms of their repair.

Following CLND completion, Zografos et al. [13] reported technical details, including internal oblique and transversus abdominis approximation with absorbable material, external oblique aponeurosis and inguinal ligament approximation with monofilament non-absorbable sutures, inguinal and Cooper’s ligament approximation with interrupted non-absorbable sutures, medial to femoral vessels and between the inguinal ligament and iliac fascia, lateral to femoral vessels. In this series of 150 pelvic groin dissections, there were no reports of inguinal ventral hernias [13]. However, a 1% frequency of incisional hernia following inguinal ligament section has also been reported [14], which may be an underestimate, as ventral inguinal hernias are late procedural complications in lethal disease states and, therefore, considered to be relatively insignificant. However, as systemic therapies improve, so will life expectancy, suggesting that increasing numbers of patients may require corrective surgery for such procedures in the future.

Our center is highly experienced in the various surgical procedures used for primary melanomas, metastatic melanomas and associated surgical complications [[15], [16], [17], [18], [19], [20]]. However, with respect to the repair of incisional inguinal ventral hernias, surgeons face particular problems that do not arise with common ventral hernias. In post-CLND incisional inguinal ventral hernias, the peritoneal sac runs along the ileo-psoas fascia and upper aspects of femoral vessels, making mesh fixation difficult and dangerous. Furthermore, the identification of correct anatomical plains is made more difficult in tissues rendered less-resistant by wound complications that result from extended lymph node dissection.

Here, we report that these difficulties can be overcome using the laparoscopic approach described. In this method, careful blunt dissection adhesiolysis provided good visualization and permitted accurate measurement of hernia margins, and an optimal repair was achieved by fixing a large intraperitoneal mesh, overlapping lesion margins and abdominal borders by at least 3 cm, at superior and lateral margins with non-absorbable fasteners and at the inferior margin by cyanoacrylate glue, creating a 5 cm overlap of femoral vessels, muscular plane and associated nerves, without compromising strength or causing vessels or nerve or injury. During this procedure, performed primarily for symptom relief and to prevent future problems, however, the hernial sac was not be removed due to tight subcutaneous adhesions in order to avoid unnecessary skin-damage, resulting in a sub-optimal aesthetic outcome. This aspect was discussed extensively with the patient prior to surgery, who provided fully informed consent.

In conclusion, incisional inguinal ventral hernias are underestimated late complications of CLND, the repair of which is challenging due to difficulty in identifying safe anatomical regions for the strong and stable anchoring of mesh, particularly at the inferior margin border. The present case study represents the first report of a laparoscopic trans-peritoneal technique that successfully repaired a large incisional inguinal ventral hernia. Technical difficulties of the procedure were surmounted, even in un-sutured conditions, by utilizing a larger mesh that extensively overlapped lesion margins and abdominal borders and by the judicial use of permanent fasteners (superior and lateral margins) and cyanoacrylate glue (inferior margins) for mesh fixation to diminish the risk of vessel and/or nerve damage.

## Sources of funding

There are no sources of funding.

## Ethical approval

Approval from the ethics committee is not required from our institution for a case report.

## Consent

Patient expressed a written informed consent to accept the publication of this paper.

## Author’s contribution

Author statement about individual contributions: Clementi M & Guadagni S: Conceptualization; Di Furia M, Sista F: Data curation; Guadagni S: Validation; Clementi M & Mackay AR: Writing - original draft-editing.

Authorship: All authors provided substantial contributions to the following: (1) the conception and design of the study; (2) drafting the article or revising it critically for important intellectual content; (3) final approval of the version to be submitted.

## Registration of research studies

Rsearchregistry: 5181.

## Guarantor

Prof. Clementi Marco.

## Provenance and peer review

Editorially reviewed, not externally peer-reviewed.

## Declaration of Competing Interest

None.
